# Correction to: Automatic reconstruction of metabolic pathways from identified biosynthetic gene clusters

**DOI:** 10.1186/s12859-021-04094-8

**Published:** 2021-04-19

**Authors:** Snorre Sulheim, Fredrik A. Fossheim, Alexander Wentzel, Eivind Almaas

**Affiliations:** 1 Department of Biotechnology and Food Science, NTNU - Norwegian University of Science and Technology, Sem Sælands vei 8, 7034 Trondheim, Norway; 2 Department of Biotechnology and Nanomedicine, SINTEF Industry, Richard Birkelands vei 3, 7034 Trondheim, Norway; 3 K.G. Jebsen Center for Genetic Epidemiology, NTNU - Norwegian University of Science and Technology, Håkon Jarls gate 11, 7030 Trondheim, Norway

## Correction to: Sulheim et al. BMC Bioinformatics (2021) 22:81 10.1186/s12859-021-03985-0

Following the publication of the original article [1], the authors identified that the legends were placed incorrectly in Additional files 1 and 2. The legends are now placed in the correct files.

The original article [1] has been corrected.
