# Supplementary material for: Automatic reconstruction of metabolic pathways from identified biosynthetic gene clusters
Source: BMC Bioinformatics. 2021 Feb 23;22:81. doi: 10.1186/s12859-021-03985-0 (PMC7901079; doi:10.1186/s12859-021-03985-0)
Supplement: Supplementary file 1 — Additional file 1. Details on tailoring reactions and synthesis of the rare extender unit methoxymalonyl-ACP, as well as a description of the analysis used to develop the heuristics that indicate the presence of these reactions from smCOG annotations. [file 12859_2021_3985_MOESM1_ESM.pdf]

Supplemental Material 1

Automatic reconstruction of metabolic pathways from identified biosynthetic gene clusters

Snorre Sulheim, Fredrik A. Fossheim, Alexander Wentzel and Eivind Almaas

Tailoring reactions

We surveyed gene clusters in MIBiG [1] to explore how specific smCOG gene annotations could be used to determine the tailoring reactions associated with the biosynthesis of a polyketide or non-ribosomal peptide synthetase: we compared the presence of specific secondary metabolism Clusters of Orthologous Groups (smCOGs) [2] annotations with the presence of different tailoring reactions as described in the literature. This led to three fairly robust heuristic that were implemented into the BiGMeC pipeline (Table S1). The data that these heuristics are based on are further described in separate sections below.

Table S1: Boolean logic used to determine tailoring reactions from smCOG gene annotation. The peptide or polyketide that is modified by the tailoring reactions is denoted by X_n, both prior and subsequent to the tailoring. Other abbreviations used in the table: bpg: 1,3-biphopshoglycerate; P_i: phosphate; P_{ii}: diphosphate; CoA: Coenzyme A.

smCOG logic	Reaction
1256 and 1084	bpg + NADH + H^+ + X_n → X_n + 2 P_i + NAD^+
1002 and 1109	glycine + succinyl-CoA + ATP + X_n → X_n + AMP + P_{ii} + CO_2 + H_2O + CoA
1062	glucose 6-phophate + X_n → X_n + P_i + H^+

Addition of glycerate

Tailoring by addition of glycerate from 1,3-biphopshoglycerate was identified from the presence of the smCOGs 1256 (FkbH like protein) and 1084 (3-oxoacyl-(acyl carrier protein) synthase III). Across all BGCs in MIBiG, this heuristic provides a correct tailoring reaction in 10 out of 11 cases (Table S2). The single error derives from BGC0000082 which still incorporates a glycerate unit, but lacking the smCOG 1084 annotation.

Incorporation of 2-Amino-3-hydroxycyclopent-2-enone

The tailoring reaction that incorporate 2-Amino-3-hydroxycyclopent-2-enone synthesized from succinyl-CoA and glycine [12], was identified from the

Table S2: Listing of BGCs in MIBiG used to determine when the addition of glycerate is used to tailor the polyketide or peptide. These are all BGCs in MIBiG containing the FkbH domain and adds glycerate. Of these 11 BGCs, 10 are annotated with the smCOG 1084.

MIBiG ID	Product	1084 present	Reference
BGC0000001	Abyssomycin	Yes	[3]
BGC0000036	Chlorothricin	Yes	[4]
BGC0000082	Kijanimicin	No	[5]
BGC0000133	Quartromicin	Yes	[6]
BGC0000140	4H3H2HMe2HF5	Yes	[6]
BGC0000162	Tetrocarcin	Yes	[7]
BGC0000164	Tetronomycin	Yes	[8]
BGC0001004	Lobophorin B	Yes	[9]
BGC0001183	Lobophorin A	Yes	[9]
BGC0001204	Versipelostatin	Yes	[10]
BGC0001288	Maklamicin	Yes	[11]

presence of the smCOGs of 1109 (8-amino-7-oxononanoate synthase) and a neighboring 1002 (AMP-dependent ligase and synthetase). This pair of smCOG annotations were present in 8 BGCs in MIBiG, of which 5 features a tailoring reaction that incorporates 2-Amino-3-hydroxycyclopent-2-enone (Table S3). For one of the BGCs (BGC0001420) the available literature was not sufficient to make a clear decision about the compound tailoring. The remaining two BGCs (BGC0000091 and BGC0001063) feature a similar tailoring reaction where the succinyl-CoA precursor is replaced by several malonyl-CoA units [13, 14].

Glycosylation

Glycosylation of the peptide or polyketide is identified by the presence of smCOG 1062 (glycosyltransferase), and as one might expect the number of incorporated sugar monomers increase with increasing number of smCOG 1062 annotations (Table S4). Based on 40 BGC synthesis pathways that incorporate sugar monomers (we consider Komodoquinone B as an outlier), we find a significant correlation between the number of incorporated sugar monomers and the number of smCOG 1062 Pearson $\rho = 0.78$, $P = 3e - 9$. Based on this result the BiGMeC pipeline therefore assumes that all glycosyltransferases are active, i.e. a 1 to 1 relationship between the number of smCOG 1062 and active glycosyltransferase tailoring reactions.

Table S3: Listing of BGCs in MIBiG used to determine when the peptide or polyketide is tailored by the incorporation of 2-Amino-3-hydroxycyclopent-2-enone synthesized from succinyl-CoA and glycine [12]. All of these BGCs are annotated with the smCOG 1002 and 1109 on adjacent genes. The "Correct" column indicate if these synthesis of the molecules associated with each BGC incorporates 2-Amino-3-hydroxycyclopent-2-enone.

MIBiG ID	Product	Correct	Reference
BGC0000028	Bafilomycin	Yes	[15]
BGC0000091	Marineosin	No	[13]
BGC0000187	Asukamycin	Yes	[16]
BGC0000213	Colabomycin	Yes	[17]
BGC0001063	Undecylprodigiosin	No	[14]
BGC0001298	Annimycin	Yes	[18]
BGC0001420	Myxochromide	Unknown	[19]
BGC0001740	Phthoxazolin	Yes	[20]

Table S4: Listing of BGCs in MIBiG that incorporates sugar monomers by glycosyltransferase in one of the tailoring steps. The "# in BGC" and "# in pathway" columns display the number of smCOG 1062 annotations in the BGC and the number of active glycosyltransferases in the synthesis of the associated compound, respectively.

MIBiG ID	Product	# in BGC	# in pathway	Reference
BGC0000002	Aculeximycin	5	8	[21]
BGC0000021	Apoptolidin	3	2	[22]
BGC0000033	Calicheamicin	4	4	[23]
BGC0000034	Candicidin	1	1	[24]
BGC0000035	Chalcomycin	2	1	[25]
BGC0000036	Chlorothricin	2	2	[4]
BGC0000042	Cremimycin	1	1	[26]
BGC0000052	ECO-02301	1	1	[27]
BGC0000054	Erythromycin B	2	2	[28]
BGC0000078	Inecidine	2	3	[29]
BGC0000081	Kedarcidin	2	2	[30]
BGC0000082	Kijanimicin	5	5	[5]
BGC0000085	Lankamycin	2	3	[31]
BGC0000092	Megalomicins	3	3	[32]
BGC0000096	Midecamicin	2	1	[33]
BGC0000102	Mycinamicin II	2	1	[34]
BGC0000105	Nanchangmycin	1	1	[35]
BGC0000108	Natamycin	1	1	[36]
BGC0000115	Nystatin A1	1	1	[37]
BGC0000136	Rifamycin	1	1	[38]
BGC0000141	Rubradirin	1	2	[39]
BGC0000148	A83543A	2	2	[40]
BGC0000151	Stambomycin A	1	1	[41]
BGC0000162	Tetrocarcin A	5	4	[7]
BGC0000165	Tiacumicin B	2	2	[42]
BGC0000167	Vicenistatin	1	1	[43]
BGC0000197	Aranciamycin	1	1	[44]
BGC0000198	Arenimycin A/B/C	2	2	[45]
BGC0000199	ArimetamycinA	2	3	[46]
BGC0000199	ArimetamycinB	1	3	[46]
BGC0000199	ArimetamycinC	1	3	[46]
BGC0000200	Arixanthomycin A	1	2	[47]
BGC0000203	BE-7585A	3	1	[48]
BGC0000208	Chelocardin	0	1	[49]
BGC0000210	Chromomycin A3	5	4	[50]
BGC0001183	Lobophorin	3	4	[9]
BGC0001452	Sipanmycin	2	4	[51]
BGC0001522	Auroramycin	2	4	[52]
BGC0001619	Ibomycin	6	7	[53]
BGC0001851	Komodoquinone B	0	5	[54]
BGC0002033	Spiramycin	3	4	[55]

Rare extender units

The smCOG gene annotations were also leveraged to determine if the enzymes responsible for the synthesis of the rare polyketide precursor methoxymalonyl-CoA were encoded in the BGC. This synthesis pathway was identified by the presence of smCOG 1256 (FkbH like protein) and smCOG 1095 (3-hydroxybutyryl-CoA dehydrogenase), see Table S5. This heuristic predicts the correct extender unit in 16 of the 24 relevant BGCs. The relevant BGC are selected from the MIBiG database based those that contains a gene with the smCOG 1245 and incorporates one or more rare extender unit. Seven of the 8 incorrect predictions are due to BGCs incorporating hydroxymalonyl-ACP and not methoxymalonyl-ACP, and the last error derives from BGC0000090 which incorporates methoxymalonyl-ACP, but lacks the smCOG 1095 annotation. Because we are currently not able to discriminate the synthesis of hydroxymalonyl-CoA from the synthesis of methoxymalonyl-ACP based on smCOG annotations, the BiGMeC pipeline assumes that the rare extender unit is methoxymalonyl-ACP. While this determines the incorporation of the reactions synthesising methoxymalonyl-ACP [56], the incorporation of methoxymalonyl-ACP as an extender unit only occurs if this requirement is fulfilled and methoxymalonyl-ACP is the extender unit as suggested by antiSMASH [57].

Table S5: List of BGCs in MIBiG that contain a gene with the smCOG 1245 annotation and incorporates a rare extender unit.

MIBiG ID	Product	Substrate	1095 present	Reference
BGC0000020	Actinosynnema	Hydroxymalonyl-ACP	Yes	[58]
BGC0000021	Apoptolidin	Methoxymalonyl-ACP	Yes	[22]
BGC0000028	Bafilomycin	Methoxymalonyl-ACP	Yes	[15]
BGC0000040	Concanamycin A	Methoxymalonyl-ACP	Yes	[59]
BGC0000065	Rustmicin	Methoxymalonyl-ACP	Yes	[60]
BGC0000066	Geldanamycin	Methoxymalonyl-ACP	Yes	[61]
BGC0000074	Herbimycin A	Methoxymalonyl-ACP	Yes	[38]
BGC0000078	Incednine	Methoxymalonyl-ACP	Yes	[29]
BGC0000090	Macbecin	Methoxymalonyl-ACP	No	[62]
BGC0000096	Midecamycin	Methoxymalonyl-ACP	Yes	[33]
BGC0000159	Tautomycin	Methoxymalonyl-ACP	Yes	[63]
BGC0000970	Chondrochloren A	Methoxymalonyl-ACP	Yes	[64]
BGC0001034	Pellasoren	Methoxymalonyl-ACP	Yes	[65]
BGC0001054	Xenocoumacin	Hydroxymalonyl-ACP	Yes	[66]
BGC0001059	Zwittermycin A	Hydroxymalonyl-ACP	Yes	[67]
BGC0001106	Oxazolomycin B	Methoxymalonyl-ACP	Yes	[68]
BGC0001348	JBIR-100	Methoxymalonyl-ACP	Yes	[69]
BGC0001511	Ansamitocin P-3	Methoxymalonyl-ACP	Yes	[70]
BGC0001537	Butyrolactol A	Hydroxymalonyl-ACP	Yes	[71]
BGC0001902	Bengamide	Hydroxymalonyl-ACP	Yes	[72]
BGC0001956	Miharamycin A	Hydroxymalonyl-ACP	Yes	[73]
BGC0001957	Amipurimycin	Hydroxymalonyl-ACP	Yes	[73]
BGC0002011	Ansacarbamitocin A	Methoxymalonyl-ACP	Yes	[74]
BGC0002033	Spiramycin	Methoxymalonyl-ACP	Yes	[55]

References

[1] Kautsar, S.A., Blin, K., Shaw, S., Navarro-Muñoz, J.C., Terlouw, B.R., van der Hooft, J.J., Van Santen, J.A., Tracanna, V., Suarez Duran, H.G., Pascal Andreu, V., *et al.*: Mibig 2.0: a repository for biosynthetic gene clusters of known function. Nucleic acids research **48**(D1), 454–458 (2020)

[2] Medema, M.H., Blin, K., Cimermancic, P., de Jager, V., Zakrzewski, P., Fischbach, M.A., Weber, T., Takano, E., Breitling, R.: antismash: rapid identification, annotation and analysis of secondary metabolite biosynthesis gene clusters in bacterial and fungal genome sequences. Nucleic acids research **39**(suppl_2), 339–346 (2011)

[3] Gottardi, E.M., Krawczyk, J.M., von Suchodoletz, H., Schadt, S., Mühlenweg, A., Uguru, G.C., Pelzer, S., Fiedler, H.-P., Bibb, M.J., Stach, J.E.M., Süssmuth, R.D.: Abyssomicin biosynthesis: formation of an unusual polyketide, antibiotic-feeding studies and genetic analysis. Chembiochem : a European journal of chemical biology **12**(9), 1401–1410 (2011)

[4] Jia, X.-Y., Tian, Z.-H., Shao, L., Qu, X.-D., Zhao, Q.-F., Tang, J., Tang, G.-L., Liu, W.: Genetic characterization of the chlorothricin gene cluster as a model for spirotetronate antibiotic biosynthesis. Chemistry & Biology **13**(6), 575–585 (2006)

[5] Zhang, H., White-Phillip, J.A., Melancon, r. Charles E, Kwon, H.-j., Yu, W.-l., Liu, H.-w.: Elucidation of the kijanimicin gene cluster: insights into the biosynthesis of spirotetronate antibiotics and nitrosugars. Journal of the American Chemical Society **129**(47), 14670–14683 (2007)

[6] He, H.-Y., Pan, H.-X., Wu, L.-F., Zhang, B.-B., Chai, H.-B., Liu, W., Tang, G.-L.: Quartromicin biosynthesis: Two alternative polyketide chains produced by one polyketide synthase assembly line. Chemistry & Biology **19**(10), 1313–1323 (2012)

[7] Fang, J., Zhang, Y., Huang, L., Jia, X., Zhang, Q., Zhang, X., Tang, G., Liu, W.: Cloning and characterization of the tetrocarcin a gene cluster from micromonospora chalcea nrrl 11289 reveals a highly conserved strategy for tetronate biosynthesis in spirotetronate antibiotics. Journal of Bacteriology **190**(17), 6014–6025 (2008)

[8] Demydchuk, Y., Sun, Y., Hong, H., Staunton, J., Spencer, J.B., Leadlay, P.F.: Analysis of the tetronomycin gene cluster: Insights into the biosynthesis of a polyether tetronate antibiotic. ChemBioChem **9**(7), 1136–1145 (2008)

[9] Zhang, C., Ding, W., Qin, X., Ju, J.: Genome sequencing of streptomyces olivaceus scsio t05 and activated production of lobophorin cr4 via metabolic engineering and genome mining. Marine drugs **17**(10), 593 (2019)

[10] Hashimoto, T., Hashimoto, J., Teruya, K., Hirano, T., Shin-ya, K., Ikeda, H., Liu, H.-w., Nishiyama, M., Kuzuyama, T.: Biosynthesis of versipelostatin: Identification of an enzyme-catalyzed [4+2]-cycloaddition required for macrocyclization of spirotetronate-containing polyketides. Journal of the American Chemical Society **137**(2), 572–575 (2015)

[11] Daduang, R., Kitani, S., Hashimoto, J., Thamchaipenet, A., Igarashi, Y., Shin-ya, K., Ikeda, H., Nihira, T.: Characterization of the biosynthetic gene cluster for maklamicin, a spirotetronate-class antibiotic of the endophytic micromonospora sp. NBRC 110955. Microbiological Research **180**, 30–39 (2015)

[12] Zhang, W., Bolla, M.L., Kahne, D., Walsh, C.T.: A three enzyme pathway for 2-amino-3-hydroxycyclopent-2-enone formation and incorporation in natural product biosynthesis. Journal of the American Chemical Society **132**(18), 6402–6411 (2010)

[13] Salem, S.M., Kancharla, P., Florova, G., Gupta, S., Lu, W., Reynolds, K.A.: Elucidation of final steps of the marineosins biosynthetic pathway through identification and characterization of the corresponding gene cluster. Journal of the American Chemical Society **136**(12), 4565–4574 (2014)

[14] Williamson, N.R., Simonsen, H.T., Ahmed, R.A.A., Goldet, G., Slater, H., Woodley, L., Leeper, F.J., Salmond, G.P.C.: Biosynthesis of the red antibiotic, prodigiosin, in serratia: identification of a novel 2-methyl-3-n-amyl-pyrrole (map) assembly pathway, definition of the terminal condensing enzyme, and implications for undecylprodigiosin biosynthesis in streptomyces. Molecular Microbiology **56**(4), 971–989 (2005)

[15] Nara, A., Hashimoto, T., Komatsu, M., Nishiyama, M., Kuzuyama, T., Ikeda, H.: Characterization of bafilomycin biosynthesis in *Kitasatospora setae* km-6054 and comparative analysis of gene clusters in actinomycetales microorganisms. The Journal of Antibiotics **70**(5), 616–624 (2017)

[16] Rui, Z., Petrícková, K., Skanta, F., Pospísil, S., Yang, Y., Chen, C.-Y., Tsai, S.-F., Floss, H.G., Petrícek, M., Yu, T.-W.: Biochemical and genetic insights into asukamycin biosynthesis. The Journal of biological chemistry **285**(32), 24915–24924 (2010)

[17] Petříčková, K., Pospíšil, S., Kuzma, M., Tylová, T., Jágr, M., Tomek, P., Chroňáková, A., Brabcová, E., Anděra, L., Krištůfek, V., Petříček, M.: Biosynthesis of colabomycin e, a new manumycin-family metabolite, involves an unusual chain-length factor. ChemBioChem **15**(9), 1334–1345 (2014)

[18] Kalan, L., Gessner, A., Thaker, M.N., Waglechner, N., Zhu, X., Szawiola, A., Bechthold, A., Wright, G.D., Zechel, D.L.: A cryptic polyene biosynthetic gene cluster in *Streptomyces calvus* is expressed upon complementation with a functional blda gene. Chemistry & Biology **20**(10), 1214–1224 (2013)

[19] Burgard, C., Zaburannyi, N., Nadmid, S., Maier, J., Jenke-Kodama, H., Luxenburger, E., Bernauer, H.S., Wenzel, S.C.: Genomics-guided exploitation of lipopeptide diversity in myxobacteria. ACS Chemical Biology **12**(3), 779–786 (2017)

[20] Suroto, D.A., Kitani, S., Arai, M., Ikeda, H., Nihira, T.: Characterization of the biosynthetic gene cluster for cryptic phthoxazolin a in streptomyces avermitilis. PLOS ONE **13**(1), 1–17 (2018)

[21] Rebets, Y., Tokovenko, B., Lushchyk, I., Rückert, C., Zaburannyi, N., Bechthold, A., Kalinowski, J., Luzhetskyy, A.: Complete genome sequence of producer of the glycopeptide antibiotic aculeximycin kutzneria albida dsm 43870t, a representative of minor genus of pseudonocardiaceae. BMC Genomics **15**(1), 885 (2014)

[22] Du, Y., Derewacz, D.K., Deguire, S.M., Teske, J., Ravel, J., Sulikowski, G.A., Bachmann, B.O.: Biosynthesis of the apoptolidins in *Nocardiopsis sp.* fu 40. Tetrahedron **67**(35), 6568–6575 (2011)

[23] Zhang, C., Bitto, E., Goff, R.D., Singh, S., Bingman, C.A., Griffith, B.R., Albermann, C., Phillips Jr, G.N., Thorson, J.S.: Biochemical and structural insights of the early glycosylation steps in calicheamicin biosynthesis. Chemistry & biology **15**(8), 842–853 (2008)

[24] Gil, J., Campelo-Diez, A.: Candicidin biosynthesis in streptomyces griseus. Applied Microbiology and Biotechnology **60**(6), 633–642 (2003)

[25] Ward, S.L., Hu, Z., Schirmer, A., Reid, R., Revill, W.P., Reeves, C.D., Petrakovsky, O.V., Dong, S.D., Katz, L.: Chalcomycin biosynthesis gene cluster from *Streptomyces bikiniensis*: Novel features of an unusual ketolide produced through expression of the chm polyketide synthase in streptomyces fradiae. Antimicrobial Agents and Chemotherapy **48**(12), 4703–4712 (2004)

[26] Amagai, K., Takaku, R., Kudo, F., Eguchi, T.: A unique amino transfer mechanism for constructing the β-amino fatty acid starter unit in the biosynthesis of the macrolactam antibiotic cremimycin. ChemBioChem **14**(15), 1998–2006 (2013)

[27] Zhang, W., Bolla, M.L., Kahne, D., Walsh, C.T.: A three enzyme pathway for 2-amino-3-hydroxycyclopent-2-enone formation and incorporation in natural product biosynthesis. Journal of the American Chemical Society **132**(18), 6402–6411 (2010)

[28] Zhang, H., Wang, Y., Wu, J., Skalina, K., Pfeifer, B.A.: Complete biosynthesis of erythromycin a and designed analogs using e. coli as a heterologous host. Chemistry & Biology **17**(11), 1232–1240 (2010)

[29] Takaishi, M., Kudo, F., Eguchi, T.: Biosynthetic pathway of 24-membered macrolactam glycoside incednine. Tetrahedron **64**(28), 6651–6656 (2008)

[30] Lohman, J.R., Huang, S.-X., Horsman, G.P., Dilfer, P.E., Huang, T., Chen, Y., Wendt-Pienkowski, E., Shen, B.: Cloning and sequencing of the kedarcidin biosynthetic gene cluster from streptoalloteichus sp. atcc 53650 revealing new insights into biosynthesis of the enediyne family of antitumor antibiotics. Molecular bioSystems **9**(3), 478–491 (2013)

[31] Arakawa, K., Kodama, K., Tatsuno, S., Ide, S., Kinashi, H.: Analysis of the loading and hydroxylation steps in lankamycin biosynthesis in streptomyces rochei. Antimicrobial agents and chemotherapy **50**(6), 1946–1952 (2006)

[32] Volchegursky, Y., Hu, Z., Katz, L., McDaniel, R.: Biosynthesis of the anti-parasitic agent megalomicin: transformation of erythromycin to megalomicin in saccharopolyspora erythraea. Molecular Microbiology **37**(4), 752–762 (2000)

[33] Cong, L., Piepersberg, W.: Cloning and Characterization of Genes Encoded in dTDP-D-mycaminose Biosynthetic Pathway from a Midecamycin-producing Strain, Streptomyces mycarofaciens. Acta Biochimica et Biophysica Sinica **39**(3), 187–193 (2007)

[34] Anzai, Y., Tsukada, S.-i., Sakai, A., Masuda, R., Harada, C., Domeki, A., Li, S., Kinoshita, K., Sherman, D., Kato, F.: Function of cytochrome p450 enzymes mycci and mycg in micromonospora griseorubida, a producer of the macrolide antibiotic mycinamicin. Antimicrobial agents and chemotherapy **56**, 3648–56 (2012)

[35] Liu, T., Lin, X., Zhou, X., Deng, Z., Cane, D.E.: Mechanism of thioesterase-catalyzed chain release in the biosynthesis of the polyether antibiotic nanchangmycin. Chemistry & biology **15**(5), 449–458 (2008)

[36] Aparicio, J.F., Barreales, E.G., Payero, T.D., Vicente, C.M., de Pedro, A., Santos-Aberturas, J.: Biotechnological production and application of the antibiotic pimaricin: biosynthesis and its regulation. Applied microbiology and biotechnology **100**(1), 61–78 (2016)

[37] Bruheim, P., Borgos, S.E., Tsan, P., Sletta, H., Ellingsen, T., Lancelin, J.-M., Zotchev, S.: Chemical diversity of polyene macrolides produced by streptomyces noursei atcc 11455 and recombinant strain erd44 with genetically altered polyketide synthase nysc. Antimicrobial agents and chemotherapy **48**, 4120–9 (2004)

[38] Chapter 13 - alkaloids derived from an m-c7n unit. In: Funayama, S., Cordell, G.A. (eds.) Alkaloids, pp. 219–232. Academic Press, Boston (2015)

[39] Kim, C.-G., Lamichhane, J., Song, K.-I., Nguyen, V.D., Kim, D.-H., Jeong, T.-S., Kang, S.-H., Kim, K.-W., Maharjan, J., Hong, Y.-S., Kang, J.S., Yoo, J.-C., Lee, J.-J., Oh, T.-J., Liou, K., Sohng, J.K.: Biosynthesis of rubradirin as an ansamycin antibiotic from streptomyces achromogenes var. rubradiris NRRL3061. Archives of Microbiology **189**(5), 463–473 (2007)

[40] Evans, D.A., Black, W.C.: Total synthesis of (+)-a83543a [(+)-lepicidin a]. Journal of the American Chemical Society **115**(11), 4497–4513 (1993)

[41] Song, L., Laureti, L., Corre, C., Leblond, P., Aigle, B., Challis, G.L.: Cytochrome p450-mediated hydroxylation is required for polyketide macrolactonization in stambomycin biosynthesis. The Journal of Antibiotics **67**(1), 71–76 (2014)

[42] Xiao, Y., Li, S., Niu, S., Ma, L., Zhang, G., Zhang, H., Zhang, G., Ju, J., Zhang, C.: Characterization of tiacumicin b biosynthetic gene cluster affording diversified tiacumicin analogues and revealing a tailoring dihalogenase. Journal of the American Chemical Society **133**(4), 1092–1105 (2011)

[43] Ogasawara, Y., Katayama, K., Minami, A., Otsuka, M., Eguchi, T., Kakinuma, K.: Cloning, sequencing, and functional analysis of the biosynthetic gene cluster of macrolactam antibiotic vicenistatin in *Streptomyces halstedii*. Chemistry & Biology **11**(1), 79–86 (2004)

[44] Luzhetskyy, A., Mayer, A., Hoffmann, J., Pelzer, S., Holzenkämper, M., Schmitt, B., Wohlert, S.-E., Vente, A., Bechthold, A.: Cloning and heterologous expression of the aranciamycin biosynthetic gene cluster revealed a new flexible glycosyltransferase. ChemBioChem **8**(6), 599–602 (2007)

[45] Jensen, P.R., Moore, B.S., Fenical, W.: The marine actinomycete genus salinispora: a model organism for secondary metabolite discovery. Natural product reports **32**(5), 738–751 (2015)

[46] Kang, H.-S., Brady, S.F.: Arimetamycin a: Improving clinically relevant families of natural products through sequence-guided screening of soil metagenomes. Angewandte Chemie International Edition **52**(42), 11063–11067 (2013)

[47] Kang, H.-S., Brady, S.F.: Arixanthomycins a-c: Phylogeny-guided discovery of biologically active edna-derived pentangular polyphenols. ACS chemical biology **9**(6), 1267–1272 (2014)

[48] Sasaki, E., Ogasawara, Y., Liu, H.-w.: A biosynthetic pathway for be-7585a, a 2-thiosugar-containing angucycline-type natural product. Journal of the American Chemical Society **132**(21), 7405–7417 (2010)

[49] Lukežič, T., Lešnik, U., Podgoršek, A., Horvat, J., Polak, T., Šala, M., Jenko, B., Raspor, P., Herron, P.R., Hunter, I.S., *et al.*: Identification of the chelocardin biosynthetic gene cluster from amycolatopsis sulphurea: a platform for producing novel tetracycline antibiotics. Microbiology **159**(Pt_12), 2524–2532 (2013)

[50] Menéndez, N., Nur-e-Alam, M., Braña, A.F., Rohr, J., Salas, J.A., Méndez, C.: Biosynthesis of the antitumor chromomycin a3 in *Streptomyces griseus*: Analysis of the gene cluster and rational design of novel chromomycin analogs. Chemistry & Biology **11**(1), 21–32 (2004)

[51] Malmierca, M.G., Pérez-Victoria, I., Martín, J., Reyes, F., Méndez, C., Salas, J.A., Olano, C.: New sipanmycin analogues generated by combinatorial biosynthesis and mutasynthesis approaches relying on the substrate flexibility of key enzymes in the biosynthetic pathway. Applied and Environmental Microbiology **86**(3) (2020)

[52] Yeo, W.L., Heng, E., Tan, L.L., Lim, Y.W., Ching, K.C., Tsai, D.-J., Jhang, Y.W., Lauderdale, T.-L., Shia, K.-S., Zhao, H., Ang, E.L., Zhang, M.M., Lim, Y.H., Wong, F.T.: Biosynthetic engineering of the antifungal, anti-mrsa auroramycin. Microbial Cell Factories **19**(1), 3 (2020)

[53] Robbins, N., Spitzer, M., Wang, W., Waglechner, N., Patel, D.J., O'Brien, J.S., Ejim, L., Ejim, O., Tyers, M., Wright, G.D.: Discovery of ibomycin, a complex macrolactone that exerts antifungal activity by impeding endocytic trafficking and membrane function. Cell Chemical Biology **23**(11), 1383–1394 (2016)

[54] Grocholski, T., Yamada, K., Sinkkonen, J., Tirkkonen, H., Niemi, J., Metsä-Ketelä, M.: Evolutionary trajectories for the functional diversification of anthracycline methyltransferases. ACS chemical biology **14**(5), 850–856 (2019)

[55] Karray, F., Darbon, E., Oestreicher, N., Dominguez, H., Tuphile, K., Gagnat, J., Blondelet-Rouault, M.-H., Gerbaud, C., Pernodet, J.-L.: Organization of the biosynthetic gene cluster for the macrolide antibiotic spiramycin in streptomyces ambofaciens. Microbiology **153**(12), 4111–4122 (2007)

[56] Chan, Y.A., Podevels, A.M., Kevany, B.M., Thomas, M.G.: Biosynthesis of polyketide synthase extender units. Natural product reports **26**(1), 90–114 (2009)

[57] Blin, K., Shaw, S., Steinke, K., Villebro, R., Ziemert, N., Lee, S.Y., Medema, M.H., Weber, T.: antismash 5.0: updates to the secondary metabolite genome mining pipeline. Nucleic acids research **47**(W1), 81–87 (2019)

[58] Yu, T.-W., Bai, L., Clade, D., Hoffmann, D., Toelzer, S., Trinh, K.Q., Xu, J., Moss, S.J., Leistner, E., Floss, H.G.: The biosynthetic gene cluster of the maytansinoid antitumor agent ansamitocin from *Actinosynnema pretiosum*. Proceedings of the National Academy of Sciences **99**(12), 7968–7973 (2002)

[59] Haydock, S.F., Appleyard, A.N., Mironenko, T., Lester, J., Scott, N., Leadlay, P.F.: Organization of the biosynthetic gene cluster for the macrolide concanamycin a in *Streptomyces neyagawaensis* atcc 27449. Microbiology **151**(10), 3161–3169 (2005)

[60] Mandala, S.M., Thornton, R.A., Milligan, J., Rosenbach, M., Garcia-Calvo, M., Bull, H.G., Harris, G., Abruzzo, G.K., Flattery, A.M., Gill, C.J., Bartizal, K., Dreikorn, S., Kurtz, M.B.: Rustmicin, a potent antifungal agent, inhibits sphingolipid synthesis at inositol phosphoceramide synthase. Journal of Biological Chemistry **273**(24), 14942–14949 (1998)

[61] Patel, K., Piagentini, M., Rascher, A., Tian, Z.-Q., Buchanan, G.O., Regentin, R., Hu, Z., Hutchinson, C.R., McDaniel, R.: Engineered biosynthesis of geldanamycin analogs for hsp90 inhibition. Chemistry & Biology **11**(12), 1625–1633 (2004)

[62] Hatano, K., Muroi, M., Higashide, E., Yoneda, M.: Biosynthesis of macbecin. Agricultural and Biological Chemistry **46**(6), 1699–1702 (1982)

[63] Li, W., Ju, J., Rajski, S.R., Osada, H., Shen, B.: Characterization of the tautomycin biosynthetic gene cluster fromStreptomyces spiroverticillatusUnveiling new insights into dialkylmaleic anhydride and polyketide biosynthesis. Journal of Biological Chemistry **283**(42), 28607–28617 (2008)

[64] Rachid, S., Scharfe, M., Blöcker, H., Weissman, K.J., Müller, R.: Unusual chemistry in the biosynthesis of the antibiotic chondrochlorens. Chemistry & Biology **16**(1), 70–81 (2009)

[65] Jahns, C., Hoffmann, T., Müller, S., Gerth, K., Washausen, P., Höfle, G., Reichenbach, H., Kalesse, M., Müller, R.: Pellasoren: Structure elucidation, biosynthesis, and total synthesis of a cytotoxic secondary metabolite from *Sorangium cellulosum.* Angewandte Chemie International Edition **51**(21), 5239–5243 (2012)

[66] Masschelein, J., Jenner, M., Challis, G.: Antibiotics from gram-negative bacteria: A comprehensive overview and selected biosynthetic highlights. Natural Product Reports **34** (2017)

[67] Kevany, B.M., Rasko, D.A., Thomas, M.G.: Characterization of the complete zwittermicin a biosynthesis gene cluster from bacillus cereus. Applied and Environmental Microbiology **75**(4), 1144–1155 (2009)

[68] Helfrich, E.J.N., Piel, J.: Biosynthesis of polyketides by trans-AT polyketide synthases. Natural Product Reports **33**(2), 231–316 (2016)

[69] Molloy, E.M., Tietz, J.I., Blair, P.M., Mitchell, D.A.: Biological characterization of the hygrobafilomycin antibiotic jbir-100 and bioinformatic insights into the hygrolide family of natural products. Bioorganic & medicinal chemistry **24**(24), 6276–6290 (2016)

[70] Lin, J., Bai, L., Deng, Z., Zhong, J.-J.: Effect of ammonium in medium on ansamitocin p-3 production by actinosynnema pretiosum. Biotechnology and Bioprocess Engineering **15**, 119–125 (2010)

[71] Harunari, E., Komaki, H., Igarashi, Y.: Biosynthetic origin of butyrolactol a, an antifungal polyketide produced by a marine-derived streptomyces. Beilstein journal of organic chemistry **13**, 441–450 (2017)

[72] Wenzel, S.C., Hoffmann, H., Zhang, J., Debussche, L., Haag-Richter, S., Kurz, M., Nardi, F., Lukat, P., Kochems, I., Tietgen, H., Schummer, D., Nicolas, J.-P., Calvet, L., Czepczor, V., Vrignaud, P., Mühlenweg, A., Pelzer, S., Müller, R., Brönstrup, M.: Production of the bengamide class of marine natural products in myxobacteria: Biosynthesis and structure–activity relationships. Angewandte Chemie International Edition **54**(51), 15560–15564 (2015)

[73] Romo, A.J., Shiraishi, T., Ikeuchi, H., Lin, G.-M., Geng, Y., Lee, Y.-H., Liem, P.H., Ma, T., Ogasawara, Y., Shin-ya, K., Nishiyama, M., Kuzuyama, T., Liu, H.-w.: The amipurimycin and miharamycin biosynthetic gene clusters: Unraveling the origins of 2-aminopurinyl peptidyl nucleoside antibiotics. Journal of the American Chemical Society **141**(36), 14152–14159 (2019)

[74] Li, X., Wu, X., Shen, Y.: Identification of the bacterial maytansinoid gene cluster asc provides insights into the post-pks modifications of ansacarbamitocin biosynthesis. Organic Letters **21**(15), 5823–5826 (2019)
